# Regulation of Clostridial Toxin Gene Expression: A Pasteurian Tradition

**DOI:** 10.3390/toxins15070413

**Published:** 2023-06-26

**Authors:** Bruno Dupuy

**Affiliations:** Institut Pasteur, Université Paris-Cité, UMR-CNRS 6047, Laboratoire Pathogenèse des Bactéries Anaérobies, F-75015 Paris, France; bdupuy@pasteur.fr

**Keywords:** *Clostridioides difficile*, toxins, regulation, metabolism

## Abstract

The alarming symptoms attributed to several potent clostridial toxins enabled the early identification of the causative agent of tetanus, botulism, and gas gangrene diseases, which belongs to the most famous species of pathogenic clostridia. Although *Clostridioides difficile* was identified early in the 20th century as producing important toxins, it was identified only 40 years later as the causative agent of important nosocomial diseases upon the advent of antibiotic therapies in hospital settings. Today, *C. difficile* is a leading public health issue, as it is the major cause of antibiotic-associated diarrhea in adults. In particular, severe symptoms within the spectrum of *C. difficile* infections are directly related to the levels of toxins produced in the host. This highlights the importance of understanding the regulation of toxin synthesis in the pathogenicity process of *C. difficile*, whose regulatory factors in response to the gut environment were first identified at the Institut Pasteur. Subsequently, the work of other groups in the field contributed to further deciphering the complex mechanisms controlling toxin production triggered by the intestinal dysbiosis states during infection. This review summarizes the Pasteurian contribution to clostridial toxin regulation studies.

## 1. Introduction

As for the other pathogenic clostridia, the disease associated with *Clostridioides* (formerly *Clostridium*) *difficile*, a Gram-positive spore-forming anaerobic bacterium, is strictly related to the production of potent exotoxins. *C. difficile* is the major pathogen responsible for nosocomial diarrhea in adults with disturbed gut microbiota due to broad-spectrum antibiotics. The clinical manifestations of *C. difficile* infections (CDI) may extend from mild diarrhea to severe life-threatening pseudomembranous colitis, a sometimes fatal gastrointestinal disease [1]. These symptoms are generally caused by the production of two toxins (TcdA and TcdB) that glucosylate members of the Rho family GTPases in host cells, thus inducing the disorganization of the actin cytoskeleton, cell death, and an acute inflammatory response [2,3]. Due to their glucosyltransferase activity, both toxins belong to the “large clostridial glucosylating toxins” family (LCGTs), encompassing lethal and hemorrhagic toxins (TcsL and TcsH, respectively) from *Paeniclostridium (formerly Clostridium) sordellii*, alpha-toxin (TcnA) from *Clostridium novyi* and the TpeL toxin from *Clostridium perfringens* [4]. In 1987, Wren’s group observed a relationship between the symptoms of antibiotic-associated diarrhea, the *C. difficile* strain, and its ability to produce toxins [5]. Subsequently, a correlation was observed between toxin levels and the severity of CDI [6], which was reinforced in the early 2000s with the emergence in North America and Europe of epidemic and hypervirulent *C. difficile* strains NAPI/027 [7,8]. These strains were responsible for a significant increase in CDI incidence and associated death, and synthesize higher levels of toxins A and B than non-epidemic strains. Therefore, diseases caused by *C. difficile* depend not only on the toxins produced, but also on the control of their synthesis and secretion, which is crucial in the pathogenicity process of *C. difficile*. Thus, deciphering the regulatory mechanisms of toxin production is important for understanding the complex responses triggered by *C. difficile* to the particular nutritional states encountered in the dysbiotic gut during infection.

Numerous studies have been conducted over the past 30 years to better understand the biochemical mode of action of the *C. difficile* toxins [3]. However, little was known about the regulation of *C. difficile* toxins when I joined Linc Sonenshein’s laboratory in 1995 to work in this area, mainly due to the difficulty of genetically manipulating this bacterium. Together with von Eichel-Streiber’s laboratory, we showed that the expression of toxin genes was dependent on the growth phases (i.e., inhibited during exponential growth and activated when cells enter the stationary phase) [9,10]. Moreover, we and others found that many environmental changes and growth conditions influence toxin levels, in which the nutritional signals with modified concentrations following gut dysbiosis are the most important environmental cues. Thus, it has been shown that limited concentrations of biotin, trehalose, or high amounts of short-chain fatty acids such as butyric acid in the culture medium stimulate toxin production [11,12], while rapidly metabolizable sugars like glucose or amino acids such as cysteine, proline, and branched-chain amino acids (BCAAs) significantly reduce toxin yields [9,13,14,15,16,17]. To date, several environmental stresses and nutritional signals have been reported to also control toxin gene expression [18]. This suggests that regulation of toxin production must be an essential strategy for the adaptation of *C. difficile* to the environmental conditions encountered during gut colonization and infection.

Looking for the molecular mechanisms that control *C. difficile* toxin gene expression depending on environmental signals was the major goal of my research when I joined Stewart Cole’s group at the Institut Pasteur in the early 2000s and later when I managed my own group from 2008.

### 1.1. In the Beginning, There Was the Pathogenicity Locus (PaLoc)

**Figure 1:**
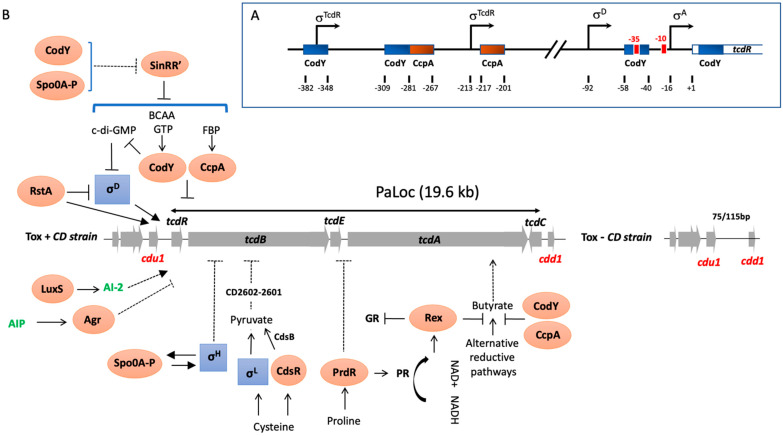
(**A**) Schematic of the promoter regions of *tcdR* denoting the relative locations of the transcriptional start sites experimentally demonstrated [9,32,34]. Blue and red boxes approximate CodY- and CcpA-binding sites within the toxin gene promoters, respectively [15,35,36]. (**B**) Direct and indirect PaLoc regulators and metabolic inputs. Activating metabolites include FBP, Fructose-1,6-bisphosphate; BCAAs, branch chain amino acids; NAD, Nicotinamide adenine dinucleotide; AI-2, auto-inducer 2, AIP, autoinducer peptide, c-di-GMP, cyclic di-guanosyl-5′monophosphate and CdsB, a cysteine desulfidase. Alternative reductive pathways include the Stickland glycine reductase (GR) pathway, succinate utilization pathway and butyrate production and square boxes correspond to alternative σ factors while oval boxes are transcriptional regulators. Arrowed lines indicate positive controls while lines ending with a bar across correspond to negative controls. Dashed arrows indicate mechanisms that are not fully understood.

One major advance in the understanding of the mechanism of toxin gene regulation came from the molecular investigation of a 19.6 kb chromosomal region known as the pathogenicity locus (Paloc) that is only found in toxigenic strains of *C. difficile* [19]. The PaLoc contains the genes encoding TcdA (*tcdA*) and TcdB (*tcdB*), and three additional accessory genes, called *tcdR*, *tcdE,* and *tcdC* (Figure 1A). In most *C. difficile* strains, the PaLoc locus is located at the same genomic position and is replaced in the non-toxigenic strains by a non-coding highly conserved 115/75 bp region [19,20]. However, we recently isolated strains with PaLoc loci integrated in different ectopic genomic sites, distant from the usual, unique Paloc integration site considered to date, suggesting that the PaLoc locus have been probably acquired by horizontal transfer [21]. Such atypical organization of the Paloc integration was reinforced in the same year by the work of Janezic et al. [22]. Except for *tcdC*, the PaLoc genes are all coordinately expressed at the entry into stationary phase [10] and we showed that the levels of *tcdA* mRNA were approximately twofold higher compared to those of *tcdB* [9]. This was in agreement with larger amounts of TcdA analyzed after toxin purification [23]. Both *tcdA* and *tcdB* are transcribed mainly from their identified promoters [9,10], while they can also be transcribed by a polycistronic transcript from an upstream promoter [5,9]. We observed that the promoter regions of these toxin genes were not similar to the canonical σ^70^ consensus promoters of prokaryotes, but rather showed strong similarities to each other, as well as to some promoters of other toxin and bacteriocin genes from several *Clostridium* species [24,25,26], the regulators involved in the transcriptional initiation of which have similarities (see below). PaLoc-like regions are conserved in *P. sordellii* [24], *C. novyi* and *C. perfringens* [27,28] containing the LCGT-encoding genes together with *tcdR*- and *tcdE*-like genes, which supports that the LCGT genes are located within PaLoc-like loci in multiple clostridia species.

### 1.2. Toxin Genes Are Specifically Transcribed by TcdR, an Alternative Sigma Factor Negatively Controlled by the Anti-Sigma Factor TcdC

Regulation of toxin synthesis is a multifactorial and complex process that allows adaptation of *C. difficile* virulence to external conditions. This currently involves several regulators and sigma factors including first those present in the PaLoc, (i.e., TcdC and TcdR), with opposite roles in toxin expression. While TcdR is a positive regulator of toxin synthesis [29,30,31,32], TcdC represses their expression [33]. The *tcdR* gene, located upstream of *tcdB* within the PaLoc (Figure 1A), encodes a small basic protein of 22 kDa, which contains a typical C-terminal helix–turn–helix (HTH) DNA-binding motif [19]. Moncrief et al. presented the first evidence that TcdR was a positive regulator of the *C. difficile* toxin genes [29] and with Linc Sonenshein’s laboratory, we showed using genetic and biochemical approaches that TcdR is required for specific transcriptional initiation of the *tcdA* and *tcdB* genes as an alternative sigma factor for RNA polymerase (RNAP) [30]. Interestingly, Ranson et al. [31] showed that a bimodal expression of toxin expression in cell is controlled by the bistability of the TcdR promoter that governs the decision between toxin-On and toxin-OFF status in a subset of cells in the population.

We demonstrated in addition that TcdR not only activates the initiation of *tcdA* and *tcdB* transcription, but also positively regulates its expression in an autoregulatory manner [32]. This was in agreement with the presence in the region upstream of the *tcdR* gene (Figure 1A) of two potential promoters with the −35 consensus sequence similar to those of the toxin gene promoters [32]. Transcription of the *tcdR* gene is not only positively controlled by TcdR, but also by SigD, a sigma factor that regulates flagellar gene expression, which is consistent with the presence of a SigD-dependent promoter in the promoter region of *tcdR* (Figure 1B) [34]. 

TcdR belongs to a new sub-group of the σ^70^ family that also encompasses other alternative σ factors of pathogenic clostridia required for the transcription of genes encoding the bacteriocin and cytotoxin of *C. perfringens* (UviA and TpeR, respectively) [25,27], the botulinum and tetanus neurotoxins (BotR and TetR, respectively) [26], the lethal and hemorrhagic toxin genes of *P. sordellii* (TcsR) [24] and the alpha-toxin (TcnA) from *C. novyi (TcnR)* [24]. While these sigma factors show similarity to the extracytoplasmic function (ECF) sigma factor family (group IV of the σ^70^-family), they differ slightly in structure and function, thus classifying them in a distinct phylogenetic sub-family of the σ^70^ family of σ factors. Moreover, we showed that TcdR-related σ factors can substitute for one another, but not for the ECF sigma factor SigW [37], supporting the idea that the TcdR-like proteins can be assigned to an unique group of σ factors (Group V) distinct from the ECF group [24,27,37]. 

The *tcdC* gene, which is located downstream of the Paloc genes on the opposite strand, is highly expressed during the exponential growth phase. However, its expression is strongly repressed at the onset of stationary growth phase, concomitantly with the transcription start of the other *tcd* genes from their own promoters in a TcdR-dependent manner [10]. In addition, we showed in vitro that expression of TcdC specifically prevents *tcdA* transcription, suggesting that TcdC is likely a negative regulator of toxin gene expression [33]. TcdC is an acidic protein with a predicted molecular weight of 26 kDa [19]. It is a membrane-associated protein [38] that is able to form dimers [33], which is consistent with a coiled-coil domain found in the central region of the protein. Such structural features support the notion that TcdC controls toxin gene transcription through modulation of TcdR activity in an anti-σ factor manner. We showed using genetic and biochemical approaches that TcdC negatively regulates *C. difficile* toxin gene expression by interfering with the ability of the TcdR-containing RNAP holoenzyme to interact with *tcdA* and *tcdB* promoters [33]. However, TcdC can also interact directly with the core RNAP, suggesting that TcdC acts by competing with TcdR to bind to the RNAP core and thereby impairs the formation of TcdR-core complexes [33]. Although these in vitro experiments clearly demonstrated that TcdC interferes with the TcdR-dependent transcription of toxin genes, other in vitro and in vivo studies have shown contradictory results on the involvement of TcdC on toxin gene expression. For instance, chromosomal complementation of the strain R20291 lacking a functional *tcdC* gene, as observed in all NAPI/027 strains, with a functional *tcdC* gene, did not change the toxin titers in vitro [39]. In addition, while *tcdC* genes are widespread among clinical isolates, the presence of *tcdC* cannot predict the hyperproduction of toxins in these strains [39,40,41]. These conflicting data may be related to the experimental variations between studies including the strains and the growth conditions used that may in part impact TcdC expression or activity. In vivo investigation of isogenic *C. difficile* strains was a prerequisite to clarifying the role of TcdC. This was performed with Dena Lyras’s group, who generated an isogenic strain of the *C. difficile* NAPI/027 strain expressing TcdC. We showed that expression of TcdC within the native host downregulates toxin production and attenuates the virulence in the hamster model of infection [42]. Further studies are still required to elucidate the role of TcdC in toxin regulation. 

### 1.3. Toxin Synthesis Is under the Control of Global Metabolic Regulators

One of the most important types of environmental signal controlling toxin production is nutritional compounds such as carbon sources or certain amino acids [9,13,43]. Overall, bacteria have developed mechanisms to uptake carbon and energy sources in the most beneficial and economical way for the cell. This regulation passes through a hierarchy of carbohydrate use. Thus, the presence of a rapidly metabolizable carbon source, such as glucose, inhibits the production of enzymes required for the transport and metabolism of other sugars. This phenomenon is called carbon catabolite repression (CCR). We showed that glucose, as well as other rapidly metabolizable carbon sources like fructose and mannitol, repress PaLoc gene expression [9]. These sugars are usually taken up via the phosphoenolpyruvate (PEP)–phosphotransferase transport system (PTS), a complex carbohydrate transport mechanism found in many Gram-positive and Gram-negative bacteria [44]. Generally, the regulation of gene transcription by such carbon sources involves the CCR system [9]. The CCR mechanism in Gram-positive bacteria, particularly well described in *Bacillus subtilis*, involve three main components. The first is called the catabolite responsive element (*cre*), a *cis*-acting DNA sequence located upstream or in the 5′ part of catabolic-regulated genes, whose modifications lead to an absence of CCR. The second component is the catabolite control protein A (CcpA), a member of the LacI/GalR family of transcriptional regulators, which in the presence of glucose, binds to the *cre* site of the catabolic-regulated genes or operons modulating their expression [45]. The third component of CCR is the phosphocarrier HPr protein phosphorylated at the regulatory residue Ser-46 (HPr-Ser46-P) by a HPr-kinase/phosphorylase, which interacts with CcpA increasing the affinity of this regulator to *cre* sites [45]. All genes encoding components of the CCR system are present in the *C. difficile* genome. Moreover, potential *cre* sites were found inside promoter regions of PaLoc genes (Figure 1B). Based on *C. difficile* mutant strains defective in the *pstI* gene of the PTS or in *ccpA*, we showed that both uptake of glucose and the global regulator CcpA are required for glucose-dependent repression of toxin genes [15]. However, we observed that the level of toxin production in the ccpA mutant grown without glucose was lower than in the parental strain, indicating that CcpA regulated other regulators involved in toxin gene transcription, such as Rex and CodY, as we showed by the transcriptomic analysis of the *ccpA* mutant [35] and below. Furthermore, we demonstrated that CcpA mediates glucose-dependent repression of toxin production by interfering directly with the promoter region or the 5′ ends of several PaLoc genes, with the strongest affinity for the promoter region of *tcdR* [35]. This is in agreement with the presence of two potential *cre* sites upstream of the transcriptional start of *tcdR* (Figure 1A; [44]). In addition, neither HPr nor HPr-Ser-64-P stimulated CcpA binding to its targets, while FBP alone did, which is somehow different from the standard mode of action of CCR in *B. subtilis*. Glucose also represses the synthesis of LCGT produced by *P. sordellii* and *C. perfringens* [24,27]. Both *P. sordellii* and *C. perfringens* encode CcpA homologs, but their role in the glucose-dependent regulation of toxin production still requires further experimental validation. A recent study showed that the ability of certain hypervirulent *C. difficile* strains, such as 027, to metabolize low levels of trehalose, a glucose disaccharide, increases disease severity through a significant increase in TcdB levels [46], although the mechanism involved is not yet known.

The PaLoc genes are transcribed in a coordinated manner according to the growth phase [10]. In *B. subtilis*, the regulator CodY monitors the nutrient sufficiency of the environment. CodY represses genes that are superfluous in nutrient-rich conditions and releases their repression when nutrients become limited in stationary phase. GTP and BCAAs, such as isoleucine and valine, act as co-repressors of CodY by increasing CodY affinity to its DNA targets [47]. Both isoleucine and valine significantly reduced *C. difficile* toxin synthesis [13]. CodY is conserved in several low-G+C Gram-positive bacteria, where it regulates not only stationary-phase genes, but also virulence factors [47]. In *C. difficile*, CodY acts as a repressor of *tcdR* gene transcription by interacting directly with its promoter region (Figure 1A), leading downstream effects on *tcdB*, *tcdE* and *tcdA* gene expression [36]. As with CcpA, in addition to its direct control of toxin gene expression, CodY also regulates master regulators such as Spo0A and metabolic pathways such as butyrate synthesis involved in toxin production [48]. Thus, regulation of toxin synthesis by both CcpA and CodY provides a molecular link between the metabolic status of the cell and *C. difficile* pathogenicity.

### 1.4. Toxin Synthesis Is also under the Control of Specific Metabolic Regulators

Among the amino acid pools modified during dysbiosis, cysteine and proline have the strongest effects on toxin production [13]. We showed that cysteine-dependent repression of toxin production is not mediated by a global nutritional regulator involved in toxin repression like Fur, CcpA or CodY, or that it acts as a reducing agent [13]. However, it requires SigL, a sigma factor belonging to the σ^54^ family primarily involved in nitrogen metabolic genes and known to play an important role in the metabolism and virulence of Gram-positive bacteria [49,50]. We have demonstrated that cysteine-dependent repression of toxin production occurs indirectly through accumulation of pyruvate, a direct by-product of cysteine catabolism controlled by SigL [17]. In contrast, addition of the pyruvate by-products, such as formate and acetate did not affect PaLoc gene transcription [17], indicating that pyruvate and sulphide, rather than cysteine, are likely the main signals modulating toxin production. This has been confirmed by the impact of the cysteine desulfidase CdsB, the main enzyme involved in the cysteine degradation whose inactivation prevent the cysteine-repression of toxin production [51]. We recently showed that the regulation of toxins by pyruvate is controlled by a two-component system (CD2602-2601) similar to the *E. coli* YpdA/YpdB TCS system [17]. The presence of proline in the medium not only represses toxin expression but also controls the major pathways of the Stickland reactions (co-fermentation of pairs of amino acids) used by *C. difficile* to produce ATP and regenerate NAD^+^ [16]. In fact, proline induces the regulator PrdR which stimulates synthesis of the proline reductase, one of the key Stickland enzymes. We have shown by global transcriptomic analysis that proline also represses through the proline reductase activity, transcription of alternative NAD^+^-generating pathways, such as the succinate utilization and butyrate production subsequently to intracellular levels of NADH/NAD+. This suggested that the regulation of toxin expression by proline was probably more related to the redox status than the direct action of PrdR. In several Gram-positive bacteria, the global redox-sensing regulator Rex directly senses changes in the redox status. Rex is only active as a DNA-binding protein when the intracellular NADH/NAD^+^ ratio is low. In a *rex* null mutant of *C. difficile*, the addition of proline did not repress fermentation pathways producing butyryl-CoA from acetyl-CoA or succinate [52]. We demonstrated that, in addition to proline-responsive expression of these alternative reductive pathways, Rex also facilitates the proline-dependent repression of toxin gene expression, probably through the regulation of butyrate known to activate toxin production [52].

### 1.5. Overall, Toxin Production Is Controlled by a Complex Regulatory Network

As of today, a large panel of regulators have been identified that control toxin production in response to the physiological lifestyle and host environmental stresses, and several reviews summarizing our current knowledge of the regulation of toxin expression have been recently published [53,54]. Among them, it is worth noting that toxin production is monitored by regulatory mechanisms established at the onset of stationary phase that also control the initiation of sporulation. Indeed, SigH, involved in the transcription of major genes of the transition phase and Spo0A, the master regulator of sporulation initiation, indirectly controls toxin gene expression at the onset of the stationary phase. While its impact is strain specific, Spo0A negatively regulates toxin production probably through regulation of *sinRR’* transcription [55,56]. In *C. difficile,* the *sin* locus encodes two regulators (SinR and SinR’) which work antagonistically to control motility, sporulation and toxin production in response to growth phase and environmental signals with a clear impact of SinR’ that negatively regulates toxin production and motility by interacting with SinR, inhibiting its influence on toxin production by a mechanism not yet known [55,56]. For SigH, we showed that it can repress toxin expression [57], presumably by coordinating the transcription of a gene encoding a repressor of toxin gene transcription such as Spo0A, since SigH is required for its expression [57]. Recently, RstA, a transcriptional regulator of the RNPP generally involved in the quorum sensing [58], was identified as a regulator positively controlling sporulation initiation and negatively impacting mobility and toxin production. It regulates toxin expression by directly binding the promoters of the toxin and *tcdR* genes, as well as the promoter for the *sigD* gene repressing expression of SigD, known to directly control *tcdR* transcription [59]. For many bacterial pathogens, virulence factors are synthesized at high cell density through quorum signaling systems. This cell–cell communication system involves the *agr* quorum-sensing locus, *agrACDB*, which is either complete (*agr2* locus) or incomplete (*agr1 locus*) according to the *C. difficile* strains. In *C. difficile* strains containing the *agr2* locus, inactivation of response regulator encoding genes *agrA* results in the decrease in toxin production presumably through the control of flagellar synthesis and the signaling molecule cyclic di-guanosyl-5′monophosphate (c-di-GMP) metabolism [60]. Indeed, an artificial increase in the intracellular levels of c-di-GMP in *C. difficile* led to switch ON/OFF expression of the *flgB* flagellar operon, including the flagellar alternative σ factor SigD, resulting in repression of *tcdR* transcription and toxin gene expression [61]. In addition to the *agr* system, the quorum sensing molecule AI-2 produced by the *luxS* gene upregulated the expression of PaLoc genes by a mechanism not yet defined [62].

Finally, the global repressor LexA and the recombinase RecA, known to control the SOS regulatory network, plays a key role in the bacterial response to DNA damage [63]. Upon DNA damage, the activated form of RecA facilitates LexA inactivation, resulting in expression of SOS genes [64]. In *C. difficile*, antibiotics known to trigger SOS responses enhanced toxin production when added at subinhibitory doses [65]. In agreement, *C. difficile* LexA not only controls DNA damage but also monitors other biological functions including regulation of toxin A production. Indeed, the production of TcdA but not TcdB increases in a *lexA* mutant compared to the wild-type strain, which is consistent with the ability of LexA to bind to the *tcdA* promoter region, containing a LexA binding motif [66]. 

## 2. Conclusions

Since the identification of the Paloc genomic region in 1986 [19], much work has been carried out, in addition to our contribution, on the regulation of *C. difficile* toxin gene expression, which appears to be highly complex, influenced by multiple environmental factors and involving a wide panel of regulators. In the murine model, we showed that toxin synthesis is expressed late during infection [67]. Thus, in vivo toxin production by clostridia must result from a complex regulatory network established along transitional phase and in response to nutrient limitation and stress during gut dysbiosis. Therefore, the mechanisms and niches associated with their toxin upregulation must also be considered as virulence factors in their own right.

## Data Availability

Not applicable.
